# Inflammatory and transcriptional roles of poly (ADP-ribose) polymerase in ventilator-induced lung injury

**DOI:** 10.1186/cc6995

**Published:** 2008-08-22

**Authors:** Je Hyeong Kim, Min Hyun Suk, Dae Wui Yoon, Hye Young Kim, Ki Hwan Jung, Eun Hae Kang, Sung Yong Lee, Sang Yeub Lee, In Bum Suh, Chol Shin, Jae Jeong Shim, Kwang Ho In, Se Hwa Yoo, Kyung Ho Kang

**Affiliations:** 1 Division of Pulmonary, Sleep and Critical Care Medicine, Department of Internal Medicine, Korea University Ansan Hospital, 516, Gojan 1-dong, Danwon-gu, Ansan 425-707, Republic of Korea; 2 Department of Nursing, College of Medicine, Pochon CHA University, 222 Yatap-dong, Bundang-gu, Sungnam 463-712, Republic of Korea; 3 Division of Respiratory and Critical Care Medicine, Department of Internal Medicine, Korea University Anam Hospital, 126-1, Anam-dong 5-ga, Seongbuk-gu, Seoul 136-705, Republic of Korea; 4 Division of Pulmonary, Allergy and Critical Care Medicine, Department of Internal Medicine, Korea University Guro Hospital, 80, Guro 2-dong, Guro-gu, Seoul 152-703, Republic of Korea; 5 Department of Clinical Pathology, College of Medicine, Kangwon National University, 26, Kangwondaehak-no, Chuncheon 200-947, Republic of Korea

## Abstract

**Introduction:**

Poly (ADP-ribose) polymerase (PARP) participates in inflammation by cellular necrosis and the nuclear factor-kappa-B (NF-κB)-dependent transcription. The purpose of this study was to examine the roles of PARP in ventilator-induced lung injury (VILI) in normal mice lung.

**Methods:**

Male C57BL/6 mice were divided into four groups: sham tracheostomized (sham), lung-protective ventilation (LPV), VILI, and VILI with PARP inhibitor PJ34 pretreatment (PJ34+VILI) groups. Mechanical ventilation (MV) settings were peak inspiratory pressure (PIP) 15 cm H_2_O + positive end-expiratory pressure (PEEP) 3 cm H_2_O + 90 breaths per minute for the LPV group and PIP 40 cm H_2_O + PEEP 0 cm H_2_O + 90 breaths per minute for the VILI and PJ34+VILI groups. After 2 hours of MV, acute lung injury (ALI) score, wet-to-dry (W/D) weight ratio, PARP activity, and dynamic compliance (C_D_) were recorded. Tumor necrosis factor-alpha (TNF-α), interleukin-6 (IL-6), myeloperoxidase (MPO) activity, and nitrite/nitrate (NO_X_) in the bronchoalveolar lavage fluid and NF-κB DNA-binding activity in tissue homogenates were measured.

**Results:**

The VILI group showed higher ALI score, W/D weight ratio, MPO activity, NO_X_, and concentrations of TNF-α and IL-6 along with lower C_D _than the sham and LPV groups (*P *< 0.05). In the PJ34+VILI group, PJ34 pretreatment improved all histopathologic ALI, inflammatory profiles, and pulmonary dynamics (*P *< 0.05). NF-κB activity was increased in the VILI group as compared with the sham and LPV groups (*P *< 0.05) and was decreased in the PJ34+VILI group as compared with the VILI group (*P *= 0.009). Changes in all parameters were closely correlated with the PARP activity (*P *< 0.05).

**Conclusion:**

Overactivation of PARP plays an important role in the inflammatory and transcriptional pathogenesis of VILI, and PARP inhibition has potentially beneficial effects on the prevention and treatment of VILI.

## Introduction

Ventilator-induced lung injury (VILI) has been established as a significant risk in patients receiving mechanical ventilation (MV). The spectrum of VILI includes not only air leaks and increases in endothelial and epithelial permeability but also increases in pulmonary and systemic inflammatory mediators [1,2]. Although the lung-protective ventilation (LPV) strategy has been shown to reduce VILI in patients with acute respiratory distress syndrome (ARDS) [3], the effectiveness of the LPV strategy may be limited because of severe spatial heterogeneity of lung involvement resulting in incomplete prevention of regional alveolar distension [4]. Alternative therapeutic strategies based on a precise understanding of its pathophysiology are necessary to completely eliminate the iatrogenic consequences of VILI.

Poly (ADP-ribose) polymerase (PARP) is a nuclear enzyme involved in the cellular response to DNA injury [5]. Upon encountering DNA strand breaks, PARP catalyzes the cleavage of nicotinamide adenine dinucleotide (NAD^+^) into nicotinamide and ADP-ribose and then uses the latter to synthesize polymers of ADP-ribose in DNA repair [6]. However, under conditions of severe DNA injury, overactivation of PARP severely depletes the intracellular stores of NAD^+^, slowing the rate of glycolysis, mitochondrial respiration, and high-energy phosphate generation, ultimately leading to cell death via the necrotic pathway [7]. This 'suicide mechanism' is closely related to the pathogenesis of disease in several pathophysiologic conditions of inflammation, and PARP inhibition or inactivation was shown to be protective against the development of inflammation due to cellular necrosis [8]. On the other hand, there is accumulating experimental evidence that suggests that PARP plays a role in nuclear factor-kappa-B (NF-κB)-dependent transcription and thus contributes to the synthesis of inflammatory mediators [9,10]. In studies of acute lung injury (ALI) by various causes, PARP was shown to play a pivotal role in the pathogenesis of lung injury and PARP inhibitors have therapeutic effects [11-14]. However, such findings have not been replicated in studies concerning the development of VILI, induced directly by an injurious ventilation strategy [15,16]. The purpose of this study was to examine the role of injurious MV strategy in PARP activation and the effects of a PARP inhibitor, in the mouse VILI model of normal lung, under the hypothesis that PARP overactivation may participate in inflammatory and transcriptional mechanisms of VILI.

## Materials and methods

### Animals and mechanical ventilation

The experimental methods were approved by the animal research committee of Korea University and the ethics committee of Korea University Medical Center. Five-week-old specific pathogen-free male C57BL/6 mice, each weighing 20 to 25 g, were randomly divided into the following four experimental groups: (a) sham tracheostomized group (sham group, n = 18); (b) LPV group (n = 18), in which the mice were ventilated with low tidal volume (V_T_) and positive end-expiratory pressure (PEEP); (c) VILI group (n = 18), in which the mice were ventilated with high V_T _without PEEP; and (d) VILI with PJ34 pretreatment group (PJ34+VILI group, n = 18), in which the mice were pretreated with the PARP inhibitor PJ34 and ventilated with the same settings as in the VILI group. Each group was subdivided into three experimental subgroups: (a) tissue subgroup (n = 6) for histopathologic examination and measurements of wet-to-dry (W/D) weight ratio and PARP activity assay; (b) bronchoalveolar lavage (BAL) subgroup (n = 6) for myeloperoxidase (MPO) activity assay and measurements of inflammatory cytokine concentration and nitric oxide (NO) metabolites in BAL fluid (BALF); and (c) tissue homogenate subgroup (n = 6) for measurement of NF-κB activity in lung tissue homogenates.

Each mouse was anesthetized with an intraperitoneal injection of 65 mg/kg of pentobarbital sodium and intubated via tracheostomy. MV was performed with a rodent ventilator (Harvard Apparatus, Holliston, MA, USA). The mice in the LPV group were ventilated with a peak inspiratory pressure (PIP) of 15 cm H_2_O, a PEEP of 3 cm H_2_O, and a respiratory rate of 90 breaths per minute. Adequate setting for the VILI model has been determined by preliminary studies using various MV settings. Histopathologic examination of the lung tissues every 30 minutes allowed determination of the time and setting that yielded typical pathological findings of VILI [17]. The typical indications developed under the following setting: PIP 40 cm H_2_O + PEEP 0 cm H_2_O + 90 breaths per minute. These changes were most prominent after about 2 hours of MV. Therefore, the VILI and PJ34+VILI groups were ventilated at this setting for 2 hours, and the LPV group mice were also ventilated for 2 hours. PIP and V_T _were measured and monitored using a linear pneumotach (series 8430; Hans Rudolph, Inc., Shawnee, KS, USA) and research pneumotach system (model RSS 100 HR; Hans Rudolph, Inc.). Changes in dynamic compliance (C_D_) between the beginning and after 2 hours of MV were calculated from the V_T_, PIP, and PEEP: C_D _= V_T_/(PIP - PEEP). To maintain deep anesthesia, half of the initial dose of pentobarbital sodium was administered after 1 hour of MV.

### Tissue preparation, wet-to-dry weight ratio, and bronchoalveolar lavage

After MV, the tissue subgroup mice were rapidly exsanguinated by dissecting the abdominal aorta. The heart and lungs were removed *en bloc *through a midsternal incision. After ligation of the left main and right upper bronchi, the left lung was excised, embedded in optimal cutting temperature compound (Tissue-Tek^®^; Sakura Finetechnical Co., Ltd., Tokyo, Japan) in a cryomold, and stored at -70°C for PARP activity assay. Excised right upper lobe was weighed in a tared container and dried in an oven until a constant weight was obtained, and the W/D weight ratio was calculated. The remnant of the right lung was immediately instilled with 4% paraformaldehyde through the right main bronchus at a hydrostatic pressure of 15 cm H_2_O and fixed in 4% paraformaldehyde for 48 hours. Paraffin blocks were prepared by dehydration with ethanol and embedding in paraffin.

For the BAL subgroup mice, the thorax was opened following euthanasia by exsanguination, and three BAL procedures were performed, each with 1 mL of phosphate-buffered saline (PBS). The retrieval fluid was centrifuged (2,000 *g *at 4°C) for 10 minutes and the supernatants were divided into aliquots and stored at -70°C until analysis for MPO activity and measurements of inflammatory cytokine concentration and NO.

### Evaluation of degree of ventilator-induced lung injury

The posterior portions of the right lower lobe were sectioned at a thickness of 5 μm, placed on glass slides, and stained with hematoxylin-eosin. A pathologist blinded to the protocol and experimental groups examined the degree of lung injury and graded the specimens by ALI score based on (a) alveolar capillary congestion, (b) hemorrhage, (c) infiltration or aggregation of neutrophils in the airspace or the vessel wall, and (d) thickness of the alveolar wall/hyaline membrane formation. Each item was graded according to the following five-point scale: 0, minimal damage; 1, mild damage; 2, moderate damage; 3, severe damage; and 4, maximal damage [18]. The degree of VILI was assessed by the sum of scores for items 0 to 16 in five randomly selected high-power fields (HPFs) (×400). The average of the sum of each field score was compared among groups.

### PARP activity assay and administration of PARP inhibitor

PARP activity in lung tissues was measured by using an immunohistochemical method of PARP activity using biotinylated NAD^+^, the substrate of the PARP [12,19]. Briefly, cryosections of 10 μm were fixed for 10 minutes in 95% ethanol at -20°C and then rinsed in PBS. Sections were permeabilized by incubation for 15 minutes at room temperature with 1% Triton X-100 in 100 mM Tris (pH 8.0). A reaction mixture consisting of 10 mM MgCl_2_, 1 mM dithiothreitol, and 30 μM biotinylated NAD^+ ^in 100 mM Tris (pH 8.0) was then applied to the sections for 30 minutes at 37°C. Reaction mixtures containing PJ34 or without biotinylated NAD^+ ^were used as controls. After three washes in PBS, incorporated biotin was detected with peroxidase-conjugated streptavidin (1:100 for 30 minutes at room temperature). After three 10-minute washes in PBS, color was developed with cobalt-enhanced nickel-DAB substrate. Sections were counterstained in Nuclear Fast Red (Vector Laboratories, Burlingame, CA, USA), dehydrated, and mounted in Vectamount (Vector Laboratories). PARP activity was quantified by summing the numbers of cells positive for PARP activity in five HPFs.

The mice in the PJ34+VILI group were intraperitoneally pretreated with 20 mg/kg of PJ34 [*N*-(6-oxo-5,6-dihydrophenanthridin-2-yl)-*N*,*N*-dimethylacetamide, hydrochloride] (Calbiochem, Darmstadt, Germany), which is not antioxidant and does not directly interfere with the reactivity of peroxynitrite [20], and there are no reports that it has an independent inhibitory effect on NF-κB. The dose was proven to be effective in lipopolysaccharide (LPS)-induced acute lung inflammation [12]. To determine optimal pretreatment time, PJ34 was administered intraperitoneally at each of 24, 12, 6, 4, 3, 2, 1, and 0.5 hours before MV to six mice at each time. The lowest PARP activity was observed after 2-hour pretreatment with PJ34. Thereafter, PARP activity increased at 1 and 0.5 hours. Therefore, the VILI+PJ34 group mice were pretreated at 2 hours before MV and the mice in the sham, LPV, and VILI groups were pretreated with 200 μL of PBS 2 hours before tracheostomy or MV.

### BALF analysis and estimation of nuclear factor-kappa-B activation in lung tissue homogenates

As an indicator of activated neutrophil accumulation, a major source of reactive oxygen species (ROS), the activity of MPO was determined directly in cell-free BALF according to the method described previously [21], with minor modifications. Tumor necrosis factor-alpha (TNF-α) and interleukin-6 (IL-6) in BALF were measured by enzyme-linked immunosorbent assay (ELISA) (R&D Systems, Minneapolis, MN, USA). Pulmonary production of NO was determined by measuring nitrate and nitrite (NO_X_), the stable end products of NO metabolism, in the BALF using an NO (NO_2_^-^/NO_3_^-^) assay kit (Assay Designs, Inc., Ann Arbor, MI, USA). Nuclear proteins from the tissue homogenate subgroup mice were prepared with a nuclear extract kit (Active Motif, Carlsbad, CA, USA). Activation of the NF-κB p65 subunit in 5 μg of nuclear extracts was measured using an NF-κB p65 ELISA-based transcription factor assay kit (TransAMTM NF-κB p65 Transcriptional Factor Assay Kit; Active Motif) [22,23].

### Statistical analysis

All data are expressed as mean ± standard error of the mean. Statistical analysis was performed using SPSS for Windows^® ^(Release 11.0.1; SPSS Inc., Chicago, IL, USA). Intergroup differences were determined by nonparametric Mann-Whitney *U *and Kruskal-Wallis tests. Statistical significance was defined as a *P *value of less than 0.05. Spearman rank correlation coefficient was used to determine the correlations between PARP activity in the tissues and the other parameters examined.

## Results

### Expression of PARP and protective effects of PJ34 in ventilator-induced lung injury

**Figure 1:**
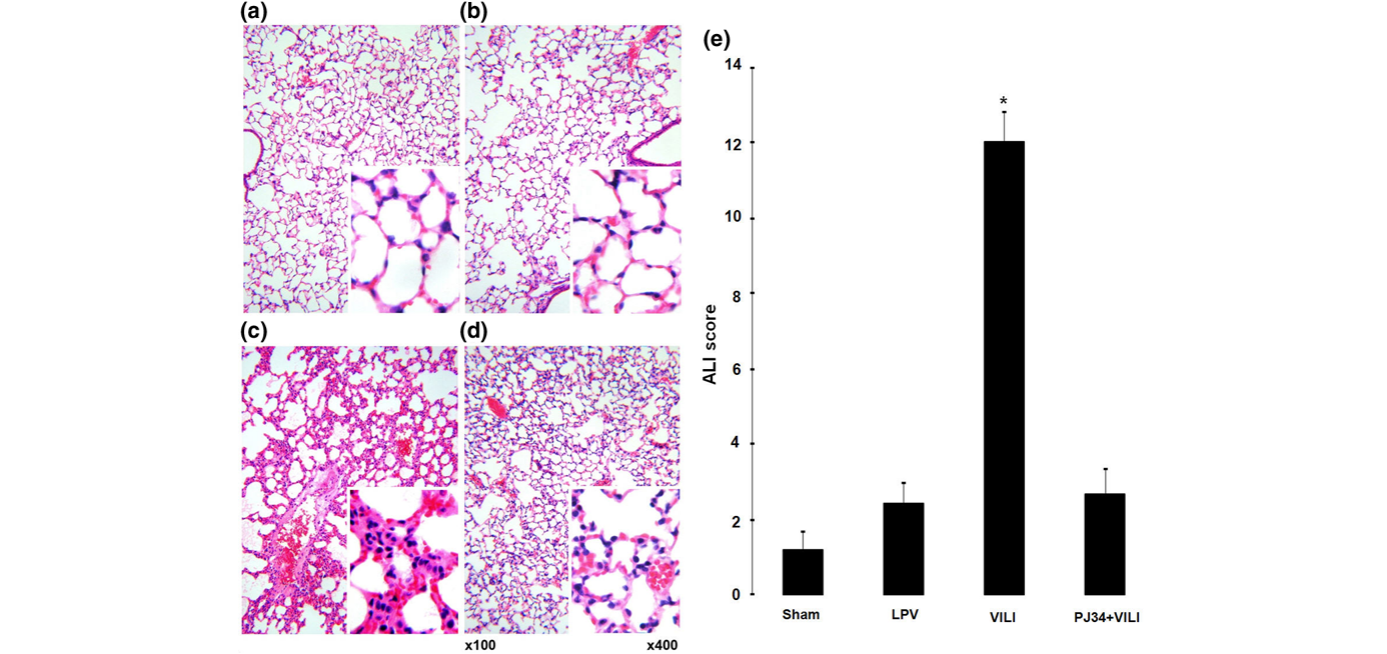
Histopathologic findings and acute lung injury (ALI) scores. The ventilator-induced lung injury (VILI) group **(c) **showed typical findings of lung injury, such as intra-alveolar exudates, hyaline membrane formation, inflammatory cell infiltration, intra-alveolar hemorrhage, and interstitial edema. These findings were markedly decreased in the PJ34+VILI group **(d)**. The sham **(a) **and lung-protective ventilation (LPV) **(b) **groups were almost normal. ALI scores **(e) **were different among the groups (*P *< 0.0001 by the Kruskal-Wallis test). The VILI group showed higher ALI scores than the other groups (*P < 0.05).

**Figure 2:**
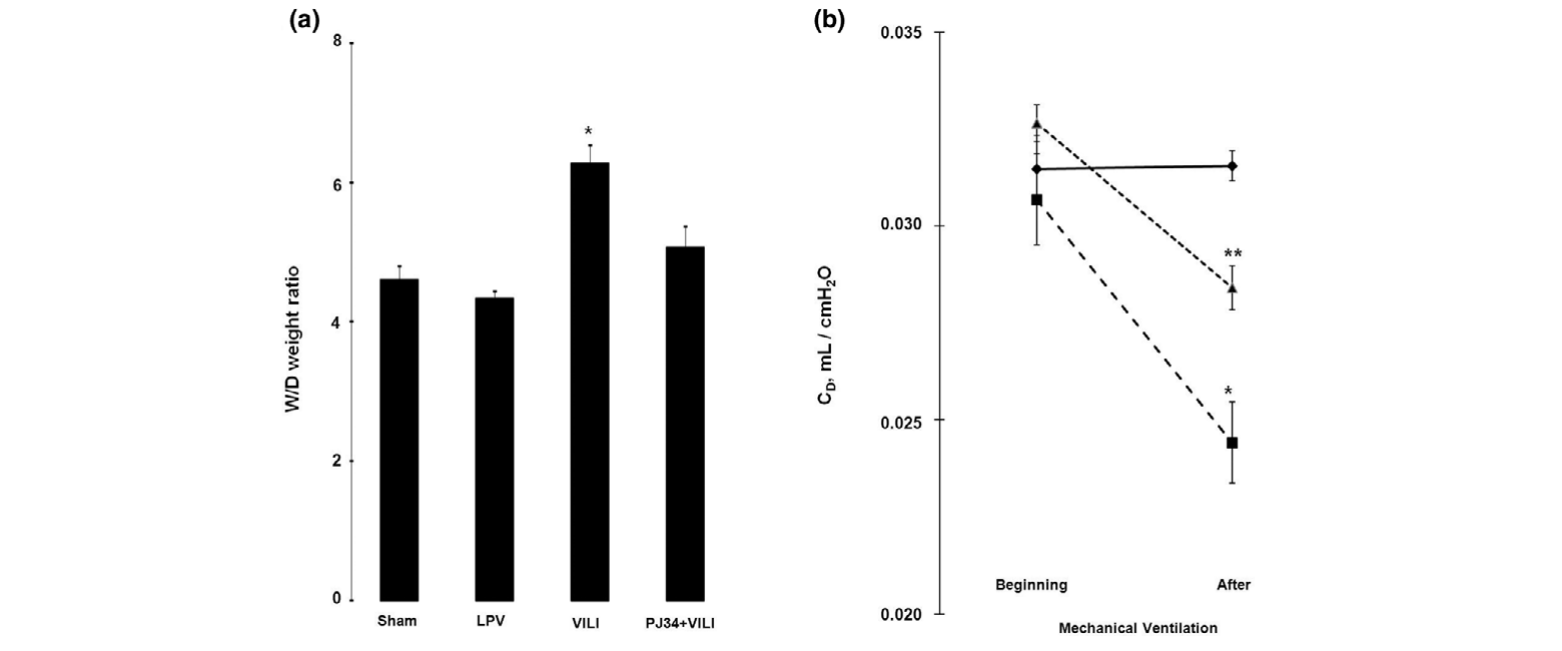
Wet-to-dry (W/D) weight ratio and dynamic compliance (C_D_). **(a) **W/D weight ratio was higher in the ventilator-induced lung injury (VILI) group than in the other groups (*P < 0.05), and the difference between all groups was significant (*P *= 0.001 by the Kruskal-Wallis test). **(b) **C_D _at the beginning of mechanical ventilation (MV) was similar among the lung-protective ventilation (LPV) (◆), VILI (■) and PJ34+VILI (▲) groups (*P *= 0.368 by the Kruskal-Wallis test). After 2 hours of MV, C_D _of the VILI group was lower than those of the other groups (*P < 0.05). C_D _of the PJ34+VILI group was higher than that of the VILI group (**P = 0.021) and lower than that of the LPV group (**P = 0.020) (*P *= 0.007 by the Kruskal-Wallis test among the three groups).

Histopathologic examination of the VILI group indicated high levels of ALI parameters (Figure 1c). These findings of lung injury were markedly reduced in the PJ34+VILI group (Figure 1d). In quantitative comparison by ALI score (Figure 1e), the VILI group (12.0 ± 0.87) showed a significantly higher score than the sham and LPV groups (1.20 ± 0.58 and 2.40 ± 0.6, respectively) (*P *< 0.05). The score of the PJ34+VILI group (2.67 ± 0.67) was significantly lower than that of the VILI group (*P *= 0.001) and was not different from those of the sham and LPV groups (*P *> 0.05). W/D weight ratio (Figure 2a) was also higher in the VILI group (6.28 ± 0.26) than in the sham and LPV groups (4.60 ± 0.21 and 4.33 ± 0.11, respectively) (*P *< 0.05). In the PJ34+VILI group, the ratio (5.05 ± 0.32) was significantly decreased relative to that of the VILI group (*P *= 0.012) and was similar to those of the sham and LPV groups (*P *> 0.05). There were no differences in C_D_s (Figure 2b) at the beginning of MV among the LPV, VILI, and PJ34+VILI groups (0.0314 ± 0.0009, 0.0307 ± 0.0012, and 0.0327 ± 0.0005 mL/cm H_2_O, respectively) (*P *= 0.368, Kruskal-Wallis test). After 2 hours of MV, however, there were statistically significant differences between the three groups (*P *= 0.007, Kruskal-Wallis test); the C_D _of the PJ34+VILI group (0.0284 ± 0.0006 mL/cm H_2_O) was significantly higher than that of the VILI group (0.0244 ± 0.0004 mL/cm H_2_O) (*P *= 0.021) and lower than that of the LPV group (0.0316 ± 0.0004 mL/cm H_2_O) (*P *= 0.020).

**Figure 3:**
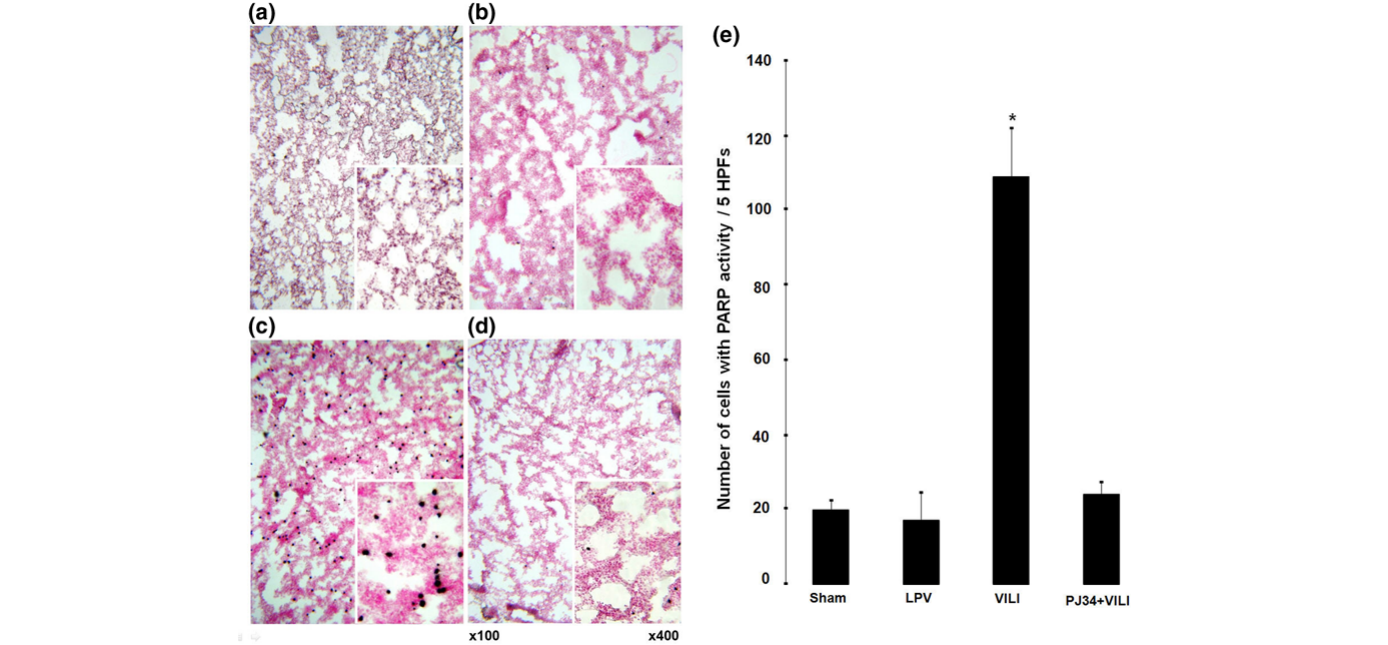
Poly (ADP-ribose) polymerase (PARP) activity assay. Larger numbers of positively stained cells were observed in the ventilator-induced lung injury (VILI) group **(c) **than in the other groups. Positive cells were almost completely absent in the sham **(a)**, lung-protective ventilation (LPV) **(b)**, and PJ34+VILI **(d) **groups. The number of cells with PARP activity **(e) **in five high-power fields (HPFs) (× 400) was higher in the VILI group (*P < 0.05) than in the other groups, with significant differences among the four groups (*P *= 0.002 by the Kruskal-Wallis test).

The PARP activity assay showed large numbers of positively stained cells in the VILI group (Figure 3c). However, in the PJ34+VILI group (Figure 3d), the number of cells was decreased markedly to the levels of the sham (Figure 3a) and LPV (Figure 3b) groups, in which positively labeled cells were almost completely absent. The number of cells with PARP activity in five HPFs (Figure 3e) in the VILI group (108.75 ± 13.185) was greater than in the sham and LPV groups (19.75 ± 2.287 and 17.00 ± 7.638, respectively) (*P *< 0.05). The number of cells in the PJ34+VILI group (23.50 ± 3.704) was lower than in the VILI group (*P *= 0.002), but there were no statistically significant differences among the sham, LPV, and PJ34+VILI groups (*P *> 0.05). In Spearman correlation analysis, PARP activity was positively correlated with the ALI score (*r *= 0.950, *P *< 0.0001) and W/D weight ratio (*r *= 0.680, *P *= 0.015) in significance. The C_D _showed negative correlation with PARP activity (*r *= -0.820, *P *= 0.002).

### Correlation of PARP activity with oxidative and nitrosative stress and the effects of PJ34

**Figure 4:**
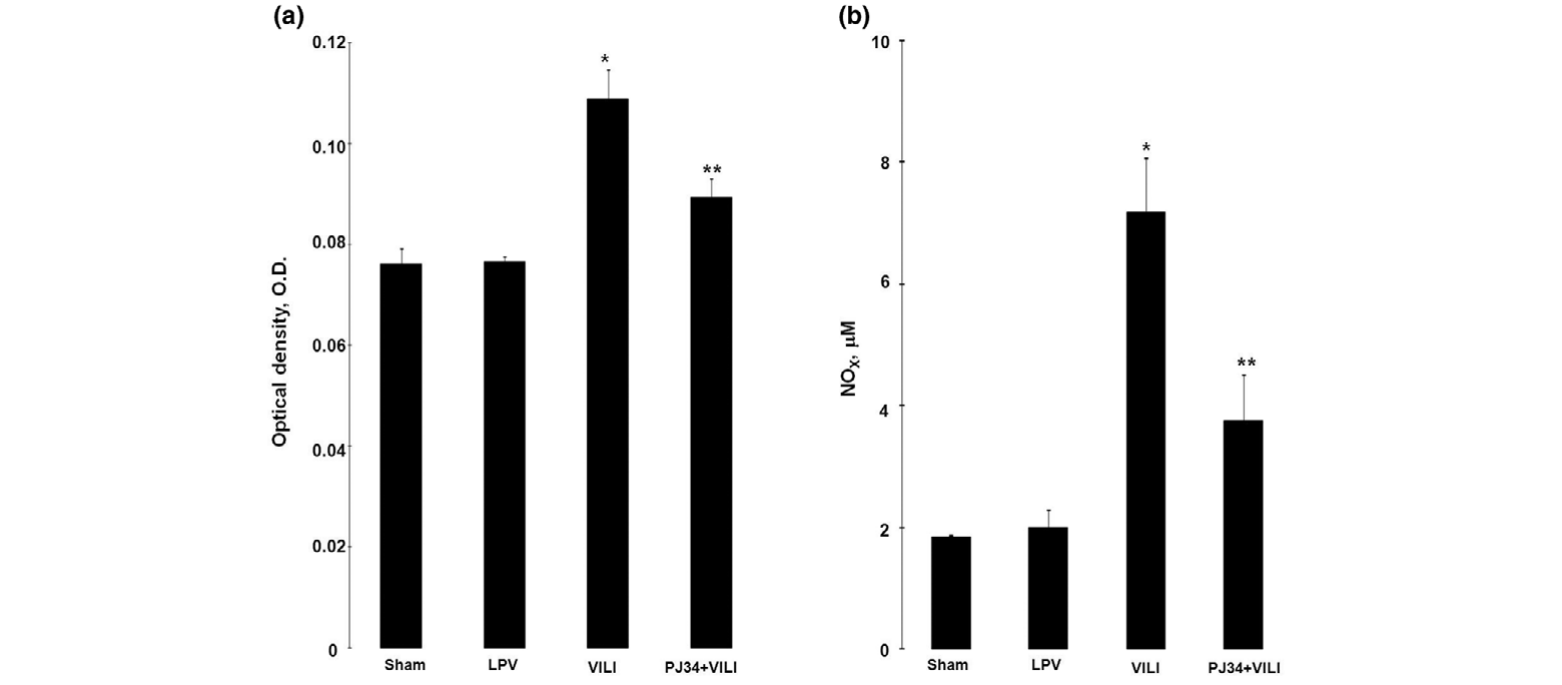
Myeloperoxidase (MPO) activity and concentration of nitric oxide (NO) metabolites. **(a) **The optical densities (ODs) of the MPO activities in the bronchoalveolar lavage fluid (BALF) were different among the groups (*P *= 0.001 by the Kruskal-Wallis test) and higher in the ventilator-induced lung injury (VILI) group than the other three groups (*P < 0.05). The PJ34+VILI group showed higher OD than the sham and lung-protective ventilation (LPV) groups (**P < 0.05). **(b) **The concentration of the NO metabolites nitrate and nitrite (NO_X_) in BALF was higher in the VILI group as compared with the other three groups (*P < 0.05). The level in the PJ34+VILI group was higher than those in the sham and LPV groups (**P < 0.05).

The optical densities (ODs) of the MPO activities in the BALF (Figure 4a) were significantly higher in the VILI group (0.109 ± 0.006 OD) than the sham (0.076 ± 0.003 OD), LPV (0.076 ± 0.001 OD), and PJ34+VILI (0.089 ± 0.004 OD) groups (*P *< 0.05). The PJ34+VILI group showed lower activity than the VILI group (*P *= 0.035) but higher activity than those of the sham and LPV groups (*P *< 0.05). Spearman correlation analysis showed this activity to be significantly correlated with PARP activity (*r *= 0.631, *P *= 0.004). The concentrations of NO metabolites nitrate and nitrite (NO_X_) in BALF (Figure 4b) were also higher in the VILI group (7.18 ± 0.9 μM) as compared with the other three groups (*P *< 0.05). The PJ34+VILI group (3.76 ± 0.76 ìM) showed lower levels than the VILI group (*P *= 0.017) and higher levels than the sham and LPV groups (1.84 ± 0.04 and 1.98 ± 0.31 μM, respectively) (*P *< 0.05). NO_X _level was also closely correlated with PARP activity (*r *= 0.523, *P *= 0.026).

### Correlations of PARP activity with inflammatory cytokines and nuclear factor-kappa-B DNA-binding activity and the effects of PJ34

**Figure 5:**
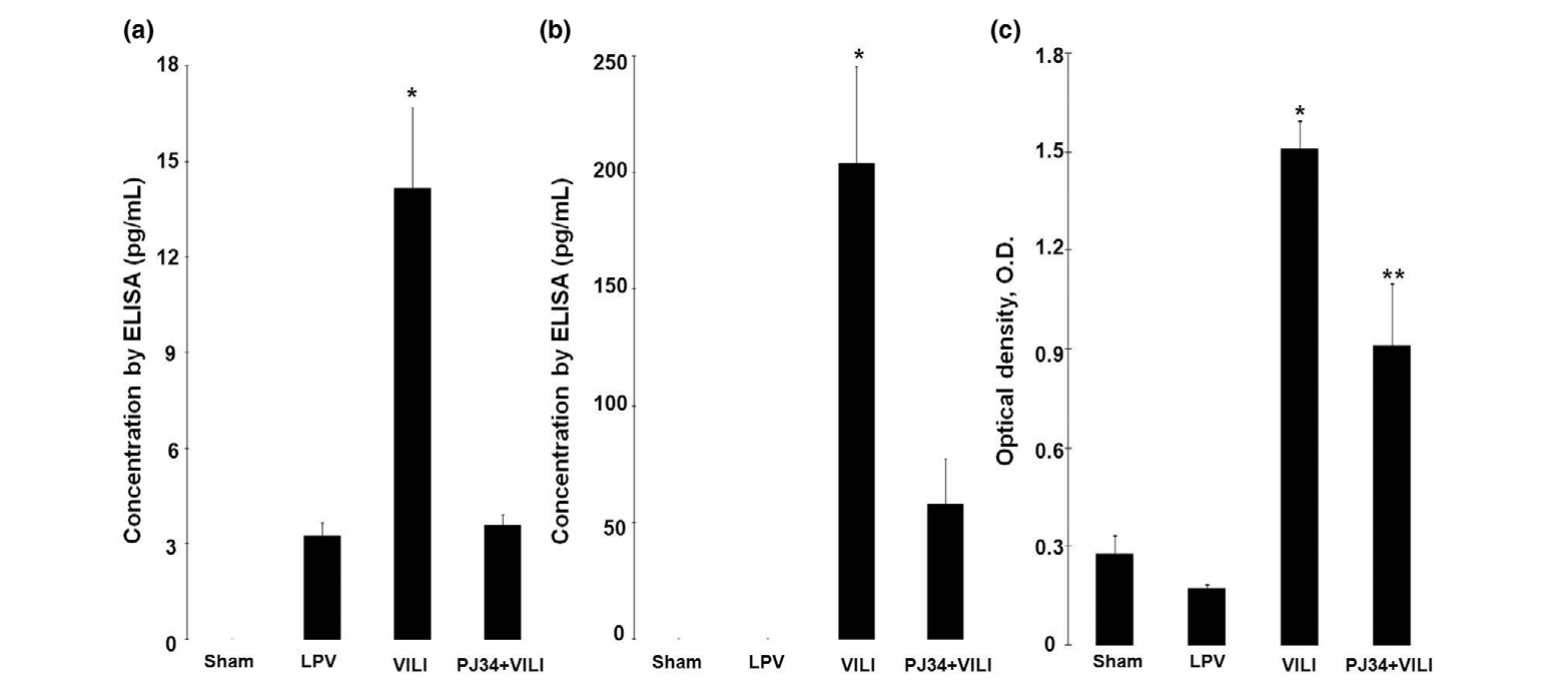
Concentrations of inflammatory cytokines and nuclear factor-κB (NF-κB) DNA-binding activity. The ventilator-induced lung injury (VILI) group showed a higher tumor necrosis factor-alpha concentration **(a) **than the lung-protective ventilation (LPV) and PJ34+VILI groups (*P < 0.05) and a higher concentration of interleukin-6 **(b) **than the PJ34+VILI group (*P = 0.015). NF-κB DNA-binding activities **(c) **in lung tissue homogenates were higher in the VILI group as compared with the other three groups (*P < 0.05). The PJ34+VILI group showed higher activity than the sham and LPV groups (**P < 0.05). ELISA, enzyme-linked immunosorbent assay.

TNF-α was not detected in BALF of the sham group, and IL-6 was not detected in the sham and LPV groups. TNF-α concentration (Figure 5a) in the VILI group (14.16 ± 2.533 pg/mL) was higher than those in the LPV and PJ34+VILI groups (3.24 ± 0.416 and 3.58 ± 0.325 pg/mL, respectively) (*P *< 0.05). IL-6 concentration (Figure 5b) in the PJ34+VILI group (57.85 ± 19.499 pg/mL) was lower than that of the VILI group (204.01 ± 41.846 pg/mL) (*P *= 0.015). The concentrations of inflammatory cytokines were correlated with PARP activity (*r *= 0.691, *P *= 0.039 for TNF-α; *r *= 0.699, *P *= 0.011 for IL-6). NF-κB DNA-binding activity measured in lung tissue homogenates (Figure 5c) was higher in the VILI group (1.51 ± 0.088 OD) than the sham and LPV groups (0.28 ± 0.056 and 0.17 ± 0.014 OD, respectively) (*P *< 0.05). However, NF-κB DNA binding in the PJ34+VILI group (0.91 ± 0.189 OD) was lower than that in the VILI group (*P *= 0.009) and higher than those in the sham and LPV groups (*P *< 0.05). NF-κB activity was positively correlated with those of PARP (*r *= 0.734, *P *= 0.001) and the inflammatory cytokines (*r *= 0.668, *P *= 0.035 for TNF-α; *r *= 0.806, *P *= 0.005 for IL-6).

## Discussion

Although the LPV strategy is useful in reducing VILI in patients with ARDS [3], it is not always possible because of highly heterogeneous lung injury in some patients [4]. To develop alternative therapeutic strategies directed at preventing VILI, it is necessary to understand the precise mechanisms involved in inflammatory reactions in lung injury. PARP, which has been known to play important roles in inflammation and NF-κB-dependent transcription, is worthy of investigation in the pathogenesis of VILI. PARP is a protein-modifying and nucleotide-polymerizing enzyme that is abundant in the nucleus and involves in DNA repair resulting from genotoxic stress by poly (ADP-ribosyl)ation [24]. However, in the case of excessive DNA damage, massive PARP activation leads to energy failure followed by necrotic cell death [24]. This mechanism and the protective effects of PARP inhibitors have also been reported to play important roles in the cases of ALI induced by LPS [12], sepsis [14], acute pancreatitis [13], bleomycin [11], burn and smoke inhalation [25], hyperoxia [26], ischemia reperfusion [27], and hyperoxia [28]. However, until recently, the role of PARP activation has not been elucidated in severe inflammatory lung injury of VILI. The present study demonstrated that PARP overactivated in the development of histopathological lung injury, pulmonary edema, and the worsening of pulmonary mechanics induced by injurious MV strategy. These changes were significantly correlated with PARP activity, and pretreatment with PARP inhibitor decreased the enzyme activity and reduced the injuries, suggesting a pivotal role of PARP in the pathogenesis of VILI. Recently, Vaschetto and colleagues [29] reported the effect of PARP inhibitor in the rat model in which MV was performed after intratracheal LPS instillation. This model is clinically relevant in studying the mechanism of ventilator-associated lung injury, which refers to the additional injury imposed on a previously injured lung by MV in either the clinical setting or experimental studies [15,16], but intratracheal administration of LPS has been reported to induce PARP overactivation in the lung tissue [12]. Therefore, this model might have limitations in examining the roles of stretch and shearing injury itself in PARP activation. It would be difficult to determine whether PARP is activated by LPS, injurious MV setting, or both and whether the PARP inhibitor exerts its effect by inhibition of PARP from LPS, injurious MV setting, or both. The primary purpose of our study was to investigate the roles of stretch and shearing forces by injurious MV in PARP activation using the VILI model of healthy animals. Through this model, we could examine and conclude that the injurious MV itself could induce the PARP activation and the PARP inhibitor could protect the injury by PARP activation, regardless of primary insult of ALI.

ROS, a major cause of lung injury, is an important trigger of DNA damage and PARP activation [8]. Although recently ROS has been reported to be produced by repetitive mechanical stretching [30-34] and shearing stresses [35-38] in cultured endothelial cells, ROS originate primarily from activated neutrophils. In the present study, oxidative stress from activated neutrophils was measured indirectly by MPO activity in BALF and the activity was increased in the VILI group and closely associated with PARP activity. In the presence of 'oxidative stress', another reactive species NO reacts rapidly with free radicals produced by activated neutrophils – superoxide – to yield peroxinitrite, a labile and toxic oxidant species and the key pathophysiologically relevant triggers of DNA single-strand breakage [39]. In the setting of ALI, airspace NO is derived primarily from the inducible form of NO synthase (NOS2), which can be induced in activated neutrophils either by stimulation with proinflammatory cytokines or by high V_T _[40]. Despite the absence of direct measurement of peroxynitrite in this experiment, the increased level of the NO_X _due to injurious MV could yield peroxynitrite by reaction with increased ROS, along with PARP activity, and inhibition of PARP reduced MPO activity and NO_X _level. Injurious MV upregulates pulmonary cytokine production, which may result in an inflammatory reaction that aggravates lung injury. Most alveolar cells are capable of producing proinflammatory mediators when stretched *in vitro *or when ventilated with a large V_T _in *ex vivo *and *in vivo *experiments [41]. On the other hand, NF-κB plays a central role as a common messenger in cytokine regulation and inflammation. In experimental models, blockage of NF-κB decreases VILI [42-44]. NF-κB activation is a critical step in the transcription of genes necessary in perpetuating the innate immune response that ultimately results in activation and extravasation of neutrophils and other immune cells, a process that starts within minutes after commencement of MV [41]. Recent studies have shown that PARP regulates the expression of various proteins at the transcriptional level. NF-κB is a key transcription factor in the regulation of this set of proteins, and PARP has been shown to act as a coactivator in NF-κB-mediated transcription and thus contributes to the synthesis of inflammatory mediators [9,10,45]. There is no consensus in the literature regarding whether the modulation of NF-κB-mediated transcription by PARP is dependent on the catalytic activity of the enzyme or its physical presence [10,46-48]. Similar to other studies, we showed that injurious MV strategies increased the concentrations of TNF-α and IL-6 in BALF and NF-κB activity in lung tissue homogenates. These changes were closely related to PARP activity. The PARP inhibitor reduced NF-κB activity and inflammatory cytokine concentrations, which were correlated with PARP activity. These results suggest the transcriptional modulation of PARP in inflammatory lung injury during VILI. To clarify whether transcriptional modulation is dependent on the catalytic activity of the enzyme or on its physical presence, experiments with PARP knockout mice are necessary. The lack of such data is a major limitation of this study, and the higher activity of NF-κB of the PJ34+VILI group than the sham and LPV groups suggests that NF-κB is activated by complex mechanisms other than PARP. Another limitation is the omission of PJ34+sham and PJ34+LPV groups. Although PJ34 has been reported to exert its effect predominantly by inhibition of PARP activity, it would be necessary to experiment with PJ34+sham and PJ34+LPV groups in order to rule out the effects of PJ34 other than PARP inhibition in the VILI model.

## Conclusion

Overactivation of PARP plays an important role in the inflammatory and transcriptional mechanisms of the pathogenesis of VILI. A clearer understanding of the action mechanisms of PARP and modulation of its effects may be clinically useful in the prevention and treatment of VILI in ARDS patients.

## Key messages

• The lung-protective ventilation strategy in acute lung injury and acute respiratory distress syndrome (ARDS) may be limited because of severe spatial heterogeneity of lung involvement. Alternative therapeutic strategies based on a precise understanding of the pathophysiology of ventilator-induced lung injury (VILI) are necessary.

• In the VILI model of normal mice lung, injurious mechanical ventilation strategy overactivated poly (ADP-ribose) polymerase (PARP), which is known to have important roles in inflammation and nuclear factor-kappa-B (NF-κB)-dependent transcription. The PARP activity was closely related with histopathologic lung injury, inflammatory cytokines, myeloperoxidase activity, nitric oxide, dynamic compliance, and NF-κB activity in the development of VILI.

• PARP inhibitor pretreatment protected the development of VILI in relation to the decrease in PARP activity.

• Understanding the roles of PARP and modulation of its effects may be clinically useful in the prevention and treatment of VILI in ARDS patients.

## Abbreviations

ALI: acute lung injury; ARDS: acute respiratory distress syndrome; BAL: bronchoalveolar lavage; BALF: bronchoalveolar lavage fluid; C_D_: dynamic compliance; ELISA: enzyme-linked immunosorbent assay; HPF: high-power field; IL-6: interleukin-6; LPS: lipopolysaccharide; LPV: lung-protective ventilation; MPO: myeloperoxidase; MV: mechanical ventilation; NAD: nicotinamide adenine dinucleotide; NF-κB: nuclear factor-kappa-B; NO: nitric oxide; NO_X_: nitric oxide metabolites nitrate and nitrite; OD: optical density; PARP: poly (ADP-ribose) polymerase; PBS: phosphate-buffered saline; PEEP: positive end-expiratory pressure; PIP: peak inspiratory pressure; ROS: reactive oxygen species; TNF-α: tumor necrosis factor-alpha; VILI: ventilator-induced lung injury; V_T_: tidal volume; W/D: wet-to-dry.

## Competing interests

The authors declare that they have no competing interests.

## Authors' contributions

JHK designed and performed the entire experiment, analyzed the data, and wrote the manuscript. MHS performed the statistical analysis and the interpretation of data. DWY and KHJ participated in the experiments and drafted the manuscript. HYK contributed to the revision of the literature search and to the drafting of the manuscript. EHK, SYL, and SYL performed the literature search. IBS advised about experimental methods. CS, JJS, KHI, and SHY reviewed the final manuscript. KHK conceived of and designed the entire study. All authors read and approved the final manuscript.
